# Complete Genome Sequence of the Ammonia-Oxidizing Bacterium *Nitrosospira* sp. Strain NRS527, Isolated from the Rhizoplane of Paddy Rice

**DOI:** 10.1128/MRA.00420-21

**Published:** 2021-07-15

**Authors:** Tatsunori Nakagawa, Yuki Tsuchiya, Reiji Takahashi

**Affiliations:** a Department of Chemistry and Life Science, College of Bioresource Sciences, Nihon University, Fujisawa, Kanagawa, Japan; University of Maryland School of Medicine

## Abstract

This work reports the complete genome sequence of the chemoautotrophic ammonia-oxidizing bacterium *Nitrosospira* sp. strain NRS527. The assembled genome is composed of a circular chromosome and two plasmids (80,750 bp and 41,389 bp, respectively).

## ANNOUNCEMENT

*Nitrosospira* sp. strain NRS527, an ammonia-oxidizing bacterium isolated from the rhizoplane of paddy rice roots (1), is an aerobic betaproteobacterium belonging to the genus *Nitrosospira* cluster 3 (2), with a tightly coiled cell shape. *Nitrosospira* is widely distributed in soil, wastewater treatment plants, freshwater environments, and marine environments (3).

A pure culture of *Nitrosospira* sp. NRS527 was obtained 20 years ago by filtering the nitrifying culture through a polycarbonate membrane (pore size, 2.0 μm) and then plating the subculture on gellan gum plates (1). For the genome analysis, *Nitrosospira* sp. NRS527 was incubated for 6 days at 30°C in 4 liters of 2-[4-(hydroxyethyl)-1-piperazinyl] ethanesulfonic acid medium (1). The nucleic acid was extracted using NucleoBond buffer set III and an AXG 500 column, following the manufacturer’s instructions (Macherey-Nagel). The genome of NRS527 was sequenced using the MiSeq (Illumina) and GridION (Oxford Nanopore Technologies) platforms. A Nextera DNA Flex library prep kit (Illumina) was used to generate a MiSeq library, and MiSeq paired-end reads were generated using MiSeq reagent kit v.2 (300 cycles; Illumina), resulting in reads with an average read length of 140 bp and 1,723,725 raw reads covering 485,881,996 sequenced bases. The quality of the Illumina read was confirmed with Sickle v.1.33 (4), using a minimum quality value score of <20 and a minimum nucleotide length of <127, resulting in 1,306,704 reads. A GridION library was prepared using the native barcoding expansion (EXP-NBD104; Oxford Nanopore Technologies) and ligation sequencing kits (Oxford Nanopore Technologies) and was sequenced with an R9.4.1 flow cell (Oxford Nanopore Technologies). Adapters of the Nanopore reads were removed using Porechop v.0.2.3 (5) (default parameters), resulting in an 18,119-bp average read length and 20,856 raw reads covering 377,886,830 sequenced bases. The quality of the Nanopore reads was assessed with Filtlong v.0.2.0 (https://github.com/rrwick/Filtlong) using a minimum nucleotide length of <1,000 to yield 235,000,000 bp and with Canu v.1.8 (6) using the “-correct” and “-nanopore-raw” parameters, resulting in 9,940 reads. The filtered Illumina and Nanopore reads were *de novo* assembled and circularized using Unicycler v.0.4.7 (7) run with default parameters and no reference, which yielded 3 contigs and a total genome size of 3,398,764 bp with a G+C content of 53.1%. An assembly graph was visualized with Bandage v.0.8.1 (8) (default parameters), showing 1 circular chromosome (3,276,625 bp) and 2 circular plasmids (80,750 bp and 41,389 bp). Genome completeness and contamination were estimated with CheckM v.1.0.12 (9) (default parameters), showing that the completeness was 100.0% and contamination was 0.24%. Subsequently, the assembled genome sequence was annotated with Prokka v.1.13 (10), which resulted in 3,044 coding sequences (CDSs) with a single copy of the 16S-23S-5S rRNA operon and 44 tRNA genes in the chromosome and with 121 of the CDSs in the plasmids.

Genes involved in oxidation of ammonia to nitrite, hydrolysis of urea, and carbon fixation were found in the chromosome.

### Data availability.

The complete genome sequence of *Nitrosospira* sp. NRS527 has been deposited in DDBJ under accession numbers AP024515, AP024516, and AP024517 and in the DDBJ Sequence Read Archive (DRA) under accession number DRA011648.
